# The prospectively-reported implementation update and score (PRIUS): a new method for capturing implementation-related developments over time

**DOI:** 10.1186/s12913-019-3927-2

**Published:** 2019-02-14

**Authors:** Edward J. Miech, Nicholas A. Rattray, Dawn M. Bravata, Barbara J. Homoya, Jennifer Myers, Teresa M. Damush

**Affiliations:** 1 VA Precision Monitoring (PRIS-M) QUERI, Richard L. Roudebush VA Medical Center, Indianapolis, Indiana USA; 2 William M. Tierney Center for Health Services Research, Regenstrief Institute, Indianapolis, Indiana USA; 3 VA Health Services Research & Development Center for Health Information and Communication (CHIC), Richard L. Roudebush VA Medical Center, Indianapolis, Indiana USA; 4 Department of Emergency Medicine, Indiana University School of Medicine, Indianapolis, Indiana USA; 5 Department of Internal Medicine and Geriatrics, Indiana University School of Medicine, Indianapolis, Indiana USA; 6 Department of Anthropology, Indiana University-Purdue University Indianapolis, Indianapolis, IN USA

**Keywords:** Implementation science, Health services research, Program evaluation, Formative evaluation, Local context

## Abstract

**Background:**

Implementation of new programs within healthcare systems can be extraordinarily complex. Individuals within the same healthcare organization often have different perspectives on how implementation of a new program unfolds over time, and it is not always clear in the midst of implementation what issues are most important or how to address them. An implementation support team within the Veterans Health Administration (VHA) sought to develop an efficient method for eliciting an ongoing, detailed and nuanced account of implementation progress from multiple viewpoints that could support and inform active implementation of two new VHA programs.

**Methods:**

The new Prospectively-Reported Implementation Update and Score (“PRIUS”) provided a quick, structured, prospective and open-ended method for individuals to report on implementation progress. PRIUS updates were submitted approximately twice a month. Responding to the prompt “What are some things that happened over the past two weeks that seem relevant from your perspective to the implementation of this project?”, individuals scored each update with a number ranging from + 3 to − 3.

**Results:**

In 2016–17, individuals submitted over 600 PRIUS updates across the two QI projects. PRIUS-based findings included that staff from different services reported fundamentally different perspectives on program implementation. Rapid analysis and reporting of the PRIUS data led directly to changes in implementation.

**Conclusions:**

The PRIUS provided an efficient, structured method for developing a granular and context-sensitive account of implementation progress. The approach appears to be highly adaptable to a wide range of settings and interventions.

## Background

Implementation of new programs within healthcare systems can be extraordinarily complex. [1] This occurs in part because implementation often relies on individuals, teams and clinical microsystems to figure out how best to put an intervention into practice within the context of a particular facility. [2] Relatively little, however, is known about how individuals, teams, and clinical microsystems interact with one another throughout the implementation process. [3] Implementation often occurs under conditions that can change rapidly and unexpectedly, and where individuals within the same healthcare organization have different perspectives on how the implementation process is unfolding. [3, 4] When implementation involves cross-functional, multidisciplinary teams − where individuals bring different expertise, experiences, priorities, and social and peer networks to implementation – team members face the additional challenge of how to integrate their different kinds of knowledge.

If there was a systematic and efficient way to collect and analyze data about how the implementation process unfolds over time, such a method could enable implementation leaders, organizational stakeholders and researchers to monitor implementation progress more closely, make revisions and course corrections as needed, assess the impact of any changes, and understand how local context influences the implementation process. In the midst of active implementation it is not always clear what issues are most important or how to address them, and a structured approach that drew independently upon the viewpoints of multiple individuals and organized their responses for rapid evaluation could identify opportunities for improving implementation on a continuous basis. Collecting data prospectively and longitudinally could also help maintain accuracy in the reporting of implementation-related information, as the ultimate success or failure of program implementation can introduce hindsight bias and retrospective evaluation can introduce recall bias. [5]

Several approaches already in use in mixed methods research and health services research precede and inform any new method concerned with the systematic capture and rapid analysis of implementation-related developments. For example, Rapid Evaluation and Assessment Methods (REAM) have long offered a way for researchers in diverse settings to collect and analyze data on an accelerated timetable while retaining rigor. [6–8] Matrix displays that integrate and organize large amounts of data in rows and columns that can be easily sorted and sifted offer a visual method to support the identification of emergent themes and findings. [9, 10] Assigning ratings (e.g., + 2 to − 2) to specific constructs from an implementation science framework like the Consolidated Framework for Implementation Research (the CFIR) provides another approach to understand how local context exerts both positive and negative influences on implementation. [4]

A major limitation across these methods in terms of their practical application is that they tend to place substantial demands on researchers and evaluation teams in terms of labor, time and resources, and thus have largely been applied retrospectively to gain insights into past episodes of implementation that have already happened. A need remains for a quick, structured, open-ended and longitudinal method that can capture and report on implementation progress that can be applied prospectively in ways that inform and influence implementation as it unfolds.

To address this gap, a team led by health services researchers who provide implementation support for ongoing projects in the national Veterans Health Administration (VHA) system in the United States sought to develop a new, efficient method for eliciting an ongoing, detailed and nuanced account of implementation progress from multiple viewpoints. This implementation team hypothesized that this new approach, called the Prospectively-Reported Implementation Update and Score (PRIUS), could generate findings and insights in a timeframe and format that supported and informed active implementation of two new VHA programs.

## Methods

### Design overview

In 2016 and 2017, the Precision Monitoring (PRIS-M) QUERI (Quality Enhancement Research Initiative) based at the Roudebush VA Medical Center in Indianapolis, Indiana was charged with supporting and studying two different quality improvement (QI) projects in VHA. The 7-person implementation team included three doctoral-level implementation scientists who collectively had been working in implementation science for over 30 years in VHA; a masters-level research nurse; a masters-level program manager; a project manager; and a research assistant. The implementation team was led by the senior implementation scientist and held regular weekly meetings, and was a formal part of the local QUERI program in Indianapolis; the QUERI program’s mission is to ensure that research gets used effectively to improve care for veterans. This research received institutional review board (IRB) approval.

The first QI project was a new “TeleSleep” program implemented in 2015–16 at the Richard L. Roudebush VA Medical Center in Indianapolis, Indiana that brought the staff of the local Sleep Medicine and Telehealth services together for the first time. The prospective one-year QI project targeted Veterans newly diagnosed with Obstructive Sleep Apnea (OSA), compared a TeleSleep protocol to usual care, and operated from October 2015 through September 2016. In the TeleSleep protocol, Roudebush VA Sleep Service staff provided Positive Airway Pressure (PAP) set up (i.e., ResMed AirSense− 10 PAP machines with wireless capability), mask fitting, and education, and the Roudebush VA Telemedicine service followed patients using a TeleSleep protocol designed to adhere to Telehealth requirements and provide state-of-the-art sleep medicine care. The second QI project was a Tele-robotics Stroke Rehabilitation QI program designed to be implemented at four pilot sites around the United States. Based at the Atlanta VA Medical Center, the Tele-Stroke Robotic Rehabilitation program provides rural Veterans who have had a stroke with an innovative, in-home solution for physical rehabilitation that improves access to care by mitigating transportation barriers for Veterans who live in rural areas at a distance from Veterans Health Administration medical centers. In FY17, the PRIS-M implementation support team assisted the Atlanta-based team during its program expansion to incorporate additional Veterans Health Administration facilities, helped to develop and refine implementation strategies, attended in-person program kick-off meetings in Atlanta, Georgia and Birmingham, Alabama and conducted baseline interviews and surveys with participating Veterans Health Administration clinicians and Veterans. As Veterans’ access to the program has continued to expand through implementation at additional Veterans Health Administration facilities, the PRIS-M implementation support team partnered with the Atlanta-based team and key stakeholders from the national Veterans Health Administration Office of Rural Health to formalize procedures, policies, practices and approaches to ensure successful implementation and larger-scale deployment across diverse Veterans Health Administration medical centers at a national level.

The core implementation strategies used in the implementation of both programs were facilitation and education. The TeleSleep program had both an executive facilitator and a coordinator facilitator, who worked in parallel to address and overcome facility-level barriers and promote implementation. The executive facilitator secured resources; conferenced with national VHA telehealth leadership; met with third-party vendors; communicated regularly with with service chiefs and facility leadership; and provided feedback to front-line clinical staff. The coordinator facilitator coordinated planning and development of the innovation; developed and coordinated the data systems including patient tracking system and CPRS templated notes; trained telehealth RNs in the TeleSleep protocols; and spanned the boundaries of traditional facility-level services (Sleep, Respiratory, Telehealth) to facilitate implementation. The executive and coordinator facilitators met together in person weekly to plan, strategize, evaluate and reflect. In the Tele-robotics program, on-site kickoffs took place where members of the national program team travelled to the participating facilities to spend a half-day training local staff on how to use the new equipment, recruit new patients with an eye to both inclusion and exclusion criteria, conduct baseline assessments, and follow new protocols.

The PRIS-M implementation support team sought to gain new insights into the implementation process for both projects and generate actionable findings that could provide specific guidance to implementation leaders regarding implementation progress and the potential need for mid-course adjustments. With these aims in mind, the team developed the new PRIUS approach and applied it to both projects.

### PRIUS development

The PRIS-M implementation support team developed a new practical tool called the Prospectively-Reported Implementation Update and Score (PRIUS) to provide a quick, structured and open-ended method for individuals to report on implementation progress. Taking between five and fifteen minutes to complete, PRIUS updates were collected approximately twice a month. PRIUS updates could come from either participants (i.e., local staff participating in program implementation) or from observers (i.e., members of the Indianapolis-based implementation support team). The two-week interval was explicitly chosen as a way to collect data with sufficient regularity without imposing undue burden on individuals reporting PRIUS updates. The format of the PRIUS was heavily influenced by the prior experience of the implementation team members from earlier projects with assigning ratings to CFIR constructs, using matrix displays, and applying rapid evaluation and assessment methods.

In the Tele-Sleep project, severe time constraints on staff participating in the program underscored the importance of finding a quick, structured, straightforward and open-ended method for participants to report on implementation progress. PRIUS was conducted as a brief verbal check-in, and began in January 2016 after the program went “live” and began to enroll patients. PRIUS sessions took place via a face-to-face or phone conversation between participants and implementation support team members (i.e., “notetakers”), depending on the preference of the participant. Participants responded verbally while notetakers prompted participants as needed and recorded responses. Participants did not need to provide anything in writing, as the notetakers wrote down all relevant data following the standardized process described below. Before conducting their first PRIUS session, members of the implementation team attended a one-hour training session in January 2016 on how to administer the PRIUS. One month later, the team met again in person to compare PRIUS entries and calibrate the scoring process so that team members shared a common understanding of valence and magnitude.

In the Tele-robotics project, individual implementation support team members independently filed their own PRIUS updates. The analytic target of the Tele-robotics project was how national implementation was progressing overall, as opposed to implementation at one specific site (as with TeleSleep).

PRIUS updates were entered into a four-column Microsoft Excel document (Table 1), with each row representing a different update. The PRIUS template was intentionally designed to capture perspectives on implementation succinctly, using a format that was both easily sortable in order to inform and guide implementation an on ongoing, iterative basis.

**Table 1:** Example of the 4-column PRIUS template populated with TeleSleep-related entries

Update	Score	Rationale	Comments
Telehealth nurse shadowed a sleep medicine physician	+1	Successful experience for nurse, who observed while sleep doc set up a new patient with PAP in Sleep Clinic	Reported that small events like this help people start to think about the notion of "TeleSleep"
TeleSleep recruitment continues to be slow	-1	Enrollment for wave 2 has been disappointing so far: 5 patients so far for March, when the goal was 30	Suggested that inertia was at play here, and that respiratory technicianss still prefer to use older machines vs. new ResMED machines
Need for new telehealth nurse	-3	Without additional staff, existing Telehealth nurses participating in TeleSleep will likely face work overload	Explained that new full time Telehealth nurse is needed to cope with future TeleSleep demand, in part because TeleSleep represents additional work above and beyond normal duties

In the first column, individuals responded to the PRIUS prompt: “What are some things that happened over the past two weeks that seem relevant from your perspective to the implementation of this project”? This structured, open-ended question was selected because it bounded responses in three specific ways: it provided a specific reporting timeframe; it engaged others by explicitly valuing their individual perspectives on implementation; and it focused attention on only the most notable (i.e., relevant) implementation developments.

In the second column, individuals scored the perceived impact of each development on the implementation of the QI project. The scoring process prompted individuals to engage in a brief reflecting and evaluating activity for each item. Individuals scored each development by selecting a number ranging from + 3 to − 3 (i.e., a 7-point scale, with zero as the middle value). Positive scores indicated a positive influence on the implementation process; negative scores indicated a negative influence; and zero indicated no discernible influence one way or the other. In terms of magnitude, 3 indicated a strong influence, 2 a moderate influence and 1 a weak influence. For example, a PRIUS update with a “+ 2” score would indicated that that the development was perceived to have a moderate positive impact on implementation of the QI project. The rationale behind the scoring process was to draw upon the experience and perspectives of individuals at the time they reported on specific implementation developments to sort each development into discrete categories of perceived impact. These numerical scores provided an additional factor with seven different possible values linked directly to the qualitative reports of implementation developments. This quantitative information provided another source of data for the implementation support team to use when sorting, analyzing, and reporting on implementation progress.

In the third column, individuals provided a brief rationale for each score. For example, a individual might explain why they scored a particular development as a “-1.”

The fourth column provided an optional space for additional comments as desired related to a particular development. This might include observed nonverbal cues like facial expressions, body language, and tone of voice; environmental factors (e.g., the PRIUS entry was based on information that came up unexpectedly during an informal phone conversation); and additional relevant details. This column was included for the notetaker to capture any important contextual details related to the reporting of specific implementation developments.

After each PRIUS session was completed, the new entries were added to a unified Microsoft Excel spreadsheet in a secure online location. This master spreadsheet featured additional columns to capture date and respondent for each entry. The growing body of PRIUS entries was then reviewed, sorted, and searched by other project team members on a shared drive, where the unified spreadsheet was available for rapid feedback, formative evaluation, and making connections between local context, implementation processes and implementation outcomes. PRIUS reviews took place approximately every two months.

In both the TeleSleep and Tele-robotics projects, implementation support team members met regularly to review the aggregated PRIUS updates to compare different perspectives on implementation-related developments, identify emergent trends, and make recommendations to implementation leaders to support ongoing implementation progress. When discrepancies in scores were observed across respondents for similar items, implementation team members flagged those implementation-related developments for further scrutiny and discussion in order to assess if the underlying source of the differences was a relatively minor semantic issue or if it reflected a deeper polarization of perspectives. If the latter, implementation team members brought these discrepancies to the attention of implementation leaders for further discussion and possible corrective action. Longitudinal analyses were conducted in two ways: by comparing PRIUS updates scored with similar values at two different timepoints (e.g., comparing all updates scored with “− 2” or “− 3” in February 2016 with “-2” or “-3” entries in April 2016); and by comparing how scores changed over time for the same kind of entry (e.g., perspectives on the quality and adequacy of the professional development provided for the program).

**Fig. 1:**
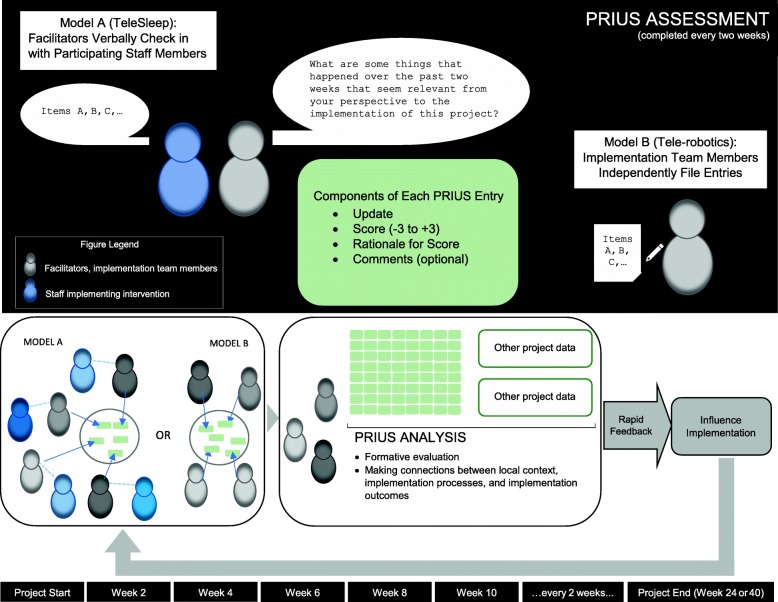
Diagram showing ongoing PRIUS data collection and analysis informing implementation over time across two projects

Figure 1 below provides a visualization of how an interconnected network of PRIUS feedback loops could be used to inform and guide implementation.

In November 2016, several months after PRIUS data collection and analysis had ended for the TeleSleep program, implementation support team members independently wrote analytic memos about their experiences using the PRIUS, including perceived strengths and limitations of the method, and then convened a face-to-face meeting to discuss their findings.

## Results

Twelve different staff members involved in the TeleSleep project participated in PRIUS sessions over a 6-month period in 2016, resulting in a total of over 190 different PRIUS updates. The Tele-robotics program generated 498 PRIUS entries related to program implementation over a 10-month period in 2017. The average PRIUS session had three or four items (or “rows” in the PRIUS template). New PRIUS updates were discussed regularly during implementation support team meetings, with PRIUS-based findings and insights shared with the lead investigators of the QI projects.

Table 2 below shows a summary of PRIUS entries from the TeleSleep and Tele-robotic projects by score and major descriptive themes. In the TeleSleep project, positive and negative interactions between staff from different clinical areas were consistently reported as having a moderate positive (+ 2) or negative (− 2) influence, respectively, on implementation. In the Tele-robotics project, strong negative (− 3) influences largely represented external developments outside the program’s direct control (e.g., crucial contracts not yet signed and executed by the Veterans Health Administration contracting office, hiring freeze impeding bringing on new staff). For both programs, initial professional development and training activities were reported as having a moderate-strong positive (+ 2 or + 3) influence; staff reluctance to participate in project implementation was reported as a weak (− 1) or moderate (− 2) negative influence; and about 1 in every 6 updates was scored as neutral (0), typically indicating the need for more time to pass before an impact could be determined.

**Table 2:** Summary of PRIUS entries from TeleSleep and Tele-robotics projects by score and major descriptive themes

TeleSleep PRIUS Updates (*n* = 190)		
Score	Frequency (%)	Major descriptive themes
+ 3	49 (26)	Positive experiences during general professional development and training events at beginning of TeleSleep project; positive interactions with vendor (ResMED); implementation of new electronic tools (e.g., TeleSleep template and tracking spreadsheet) very helpful
+ 2	28 (15)	Positive interactions between individuals from different clinical areas
+ 1	15 (8)	Small, incremental changes (e.g., enrolling an additional patient)
0	26 (14)	Potential opportunities representing a change from status quo (e.g., Telehealth not reviewing TeleSleep data during Telehealth meetings; possibility for additional funds to become available in future to TeleSleep)
−1	16 (8)	Perceived lack of interest by frontline clinical staff in starting TeleSleep program
−2	33 (17)	Negative interactions between individuals from different clinical areas; slow patient enrollment in TeleSleep between February–April 2016; perceived need for additional training to meet PAP needs of patients
−3	23 (12)	TeleSleep workload heavier than originally anticipated; distrust and hostility among individuals from different clinical areas
Tele-robotics PRIUS Updates (*n* = 498)		
+ 3	46 (9)	Optional activities completed (e.g., creating YouTube videos); face-to-face Tele-robotics kick-off events held at participating facilities; major contracts finally signed; additional full year of program funding officially approved
+ 2	99 (20)	Patient enrollment begins; Tele-robotics implementation products completed (e.g., videos, how-to guides, notes and templates); positive experiences during Tele-robotics professional development and training events
+ 1	161 (32)	Small, incremental changes (e.g., enrolling an additional patient); participation in external conferences and meetings where Tele-robotics program is brought to attention of a larger audience
0	84 (17)	On hold waiting for something to happen, like hearing back from other individuals/services not directly involved in Tele-robotics program or standing by pending completion of implementation activities still in progress
-1	66 (13)	Local staff reporting that general model for Tele-robotics program does not fit local setting and will require some modification; ongoing minor delays in getting needed signatures and approvals; target go-live dates not met
-2	33 (7)	Important clinical staff at local facilities declining to participate in Tele-robotics; negative interactions between individuals from different clinical areas within the same facility; ongoing major delays in getting necessary approvals
-3	9 (2)	External developments beyond team’s control (e.g., Contracting office not signing off on crucial paperwork, placing program in jeopardy; enterprise-wide hiring freeze; key components not included in signed contract with vendor)

Longitudinal analyses of PRIUS entries primarily identified major program-wide changes over time rather than individual-level changes. For instance, TeleSleep participants initially reported professional development and training activities as a strong positive (+ 3) in February 2016 but then reported the perceived need for additional training to meet the PAP needs of patients as a moderate negative (− 2) by April 2016. Strong positive (+ 3) influences reported in Tele-robotics corresponded initially most often to items that had been planned and successfully executed by the program (e.g., kick-off meetings at facilities, completing YouTube videos) but six months later mostly related to enterprise-level administrative actions (e.g., additional year of program funding approved, key equipment and service contracts finally signed).

Rapid analysis and reporting of the PRIUS data led directly to changes in implementation. In the TeleSleep project, for example, findings from the onset of data collection in February 2016 revealed that staff from different clinical areas reported fundamentally different perspectives on project implementation. The implementation support team shared this result with the TeleSleep implementation leader in a formally scheduled meeting to review the PRIUS data in February 2016. Reflection on these findings led to the realization that while managers and service chiefs related to sleep medicine and telehealth had regularly attended TeleSleep meetings, several key frontline staff could not make those meetings because of conflicting clinical duties. Key frontline staff from sleep medicine and telehealth, furthermore, did not know each other personally, and did not know what happened when a patient transferred from one service to the other when enrolled in the TeleSleep program.

In March 2016 patient enrollment in TeleSleep nearly ground to a halt, with only 5 patients enrolled when the original target had been 30. As a direct result of the earlier PRIUS discussion, the implementation leader together with the implementation support team decided to host a voluntary, catered “appreciation” lunch for all interested TeleSleep staff in early April 2016. As part of this lunch, the implementation leader would thank staff personally for their participation in the program and share TeleSleep data demonstrating how effective the program had been to date in terms of improving patient outcomes. The appreciation lunch was well-attended and positively received, with frontline staff from the two services engaging in personal conversations for the first time. Program recruitment by clinical staff improved so dramatically after this lunch that within a month, enrollment had to be temporarily suspended until the TeleSleep staff could catch up with the new patients. PRIUS updates collected after the lunch clearly identified this event as a turning point in program implementation, with several individuals independently reporting that it was not until this event that they understood how the TeleSleep program worked overall, the positive impact they were having on patients through the program, and the perspectives of other staff involved in the program from different service areas. As originally intended, the PRIUS enabled the implementation leader of TeleSleep to monitor implementation progress more closely, to make a needed course correction, and assess the impact of that intervention.

Several months after PRIUS data collection and analysis had ended for the TeleSleep program, implementation team members wrote analytic memos and convened an in-person meeting and group discussion in November 2016 to reflect on their experiences on using the PRIUS. The main strengths identified were that the structured but open-ended PRIUS approach helped implementation team members establish rapport quickly with participants, and that participants seemed eager to share their perspectives on implementation. In fact, there were no refusals; rather, participants were universally willing to talk with the implementation team. Several participants reported that they actively looked forward to new PRIUS sessions so they could share the latest updates from their vantage point. One nurse in the TeleSleep program even contacted the implementation team to request the initiation of PRIUS sessions when they had not yet been scheduled with that individual. Participants were also not shy about reporting developments they viewed as negative (i.e., − 2 or − 3); the PRIUS appeared to create a safe space for venting negative concerns in a constructive way in an organizational context where individuals might otherwise not feel comfortable divulging critical remarks or observations. The biggest reported limitation was that numerical scoring of PRIUS items on a + 3 to − 3 scale occasionally felt forced for participants; this awkwardness was sometimes alleviated by first introducing the + 3 to − 3 scale to participants but then using terms (positive/neutral/negative, weak/moderate/strong) rather than numbers when scoring items.

## Discussion

The PRIUS mapped directly onto several implementation strategies listed in the Expert Recommendations for Implementing Change project, including audit and feedback; facilitation; purposely reexamining the implementation; tailoring strategies; and using implementation advisors [11]. Furthermore, PRIUS data and analysis directly led to course corrections that can likewise be viewed in terms of implementation strategies: the TeleSleep “appreciation lunch” combined the two discrete implementation strategies of audit and feedback with organizing a clinician implementation team meeting. In the context of these two QI projects, the PRIUS can be understood not only as a rapid-cycle evaluation method but also as an integrated set of implementation strategies.

No special software is required to collect, organize or analyze PRIUS entries. By generating succinct descriptions of implementation-related developments along with their corresponding scores, the PRIUS offers implementation leaders, stakeholders, and researchers a pragmatic approach for monitoring how implementation unfolds in specific healthcare settings and for making mid-course adjustments as needed.

This study had several limitations. It involved only two implementation projects in Veterans Health Administration, and may not generalize to use in other projects or in other healthcare systems. The amount of time required to capture PRIUS updates was not formally tracked and cost data were not formally collected. The PRIUS method has not yet been formally validated against patient outcomes or other measures of implementation success. The PRIUS may be more feasible when applied to pilot projects when the total number of participants and stakeholders remain relatively small. We recommend future evaluations of the method include assessments of its validity and limitations, as well as its cost and time requirements.

## Conclusions

PRIUS provided an efficient, structured tool to elicit and capture perspectives of diverse participants on local implementation-related developments over time. The method turned out to be straightforward, quick and interactive, capturing valuable implementation-related data that might otherwise prove evanescent. The PRIUS allowed for the development of an ongoing, detailed and nuanced account of implementation progress that could support and inform active implementation of two diverse programs in healthcare settings.

The Veterans Health Administration PRIS-M QUERI in Indianapolis has since applied PRIUS to additional projects and found the tool to be highly adaptable to a wide range of settings. As a prospective, longitudinal and standardized approach to capturing and analyzing implementation-related developments over time, PRIUS offers a new way to develop a granular, context-sensitive account of implementation progress. Ongoing collection, review and analysis of PRIUS entries can result in new insights and actionable recommendations that support the dynamic and complex process of implementation within healthcare settings.

## Abbreviations

CFIR: Consolidated Framework for Implementation Research
CHIC: Center for Health Information and Communication
IRB: Institutional Review Board
OSA: Obstructive Sleep Apnea
PAP: Positive Airway Pressure
PRIS-M: Precision Monitoring
PRIUS: Prospectively-Reported Implementation Update and Score
QI: Quality Improvement
QUERI: Quality Enhancement Research Initiative
REAM: Rapid Evaluation and Assessment Methods
VHA: Veterans Health Administration
